# Capsaicin Rich Low-Fat Salad Dressing: Improvement of Rheological and Sensory Properties and Emulsion and Oxidative Stability

**DOI:** 10.3390/foods12071529

**Published:** 2023-04-04

**Authors:** Esra Avci, Zeynep Hazal Tekin-Cakmak, Muhammed Ozgolet, Salih Karasu, Muhammed Zahid Kasapoglu, Mohamed Fawzy Ramadan, Osman Sagdic

**Affiliations:** 1 Faculty of Chemical and Metallurgical Engineering, Department of Food Engineering, Davutpasa Campus, Yildiz Technical University, 34220 Istanbul, Turkey; 2 Istanbul Teknokent, Cerrahpasa Avcılar Campus, Istanbul University, 34320 Istanbul, Turkey; 3 Department of Clinical Nutrition, Faculty of Applied Medical Sciences, Umm Al-Qura University, Makkah 21955, Saudi Arabia

**Keywords:** hot pepper seed oil, dihydrocapsaicin, antioxidants, fat replacers, oxidation kinetics, rheology

## Abstract

This study aimed to investigate the potential use of cold-pressed hot pepper seed oil by-product (HPOB) in a low-fat salad dressing to improve its rheological properties, emulsion, and oxidative stability. The total phenolic content (TPC), the 2,2-Diphenyl-1-picrylhydrazyl (DPPH) radical scavenging activity, and CUPRIC reducing antioxidant capacity (CUPRAC) values were 317.4 mg GAE/100 g, 81.87%, and 6952.8 mg Trolox/100 g, respectively. The capsaicin, dihydrocapsaicin, and total carotenoid content were 175.8 mg/100 g, 71.01 mg/100 g, and 106.3 µg/g, respectively. All emulsions indicated shear-thinning, viscoelastic solid-like behavior, and recoverable characteristics, which were improved via enrichment with HPOB. The thermal loop test showed that the low-fat sample formulated with 3% HPOB indicated little change in the G* value, showing that it exhibited high emulsion stability. The induction period values (IP) of the salad dressing samples containing HPOB (between 6.33 h and 8.33 h) were higher than the IP values of the control samples (3.20 h and 2.58 h). The enrichment with HPOB retarded the formation of oxidative volatile compounds of hexanal, nonanal, and 1-octene-3-ol. According to the results presented in this study, HPOB could be effectively used in a low-fat salad dressing to enhance its rheological characteristics and oxidative stability.

## 1. Introduction

Salad dressing is one of the most preferred food items consisting of vegetable flavoring ingredients (sweetener, salt, garlic) and emulsifying agents (crystal inhibitors, texture enhancers, and stabilizers), as well as acidic constituents (maleic acid, acetic acid, and citric acid). Recently, consumers’ interest in salad dressing has increased. Its high oil content is the most prominent feature of salad dressing (at least 30%). Due to the trend of health-consciousness and increase in awareness of the health risk of excessive fat consumption such as cardiovascular diseases, cognitive dysfunction, diabetes, and obesity, the consumption of reduced-fat food items has increased [1,2]. Therefore, food manufacturers and scientists are trying to find new ways to produce low-fat and low-calorie foods without losing quality [3,4]. As a result, several fat substitutes are now utilized in salad dressing formulations to reduce the negative qualities of the finished product that occur when fat is reduced or removed.

Fat replacers are substances that could mimic the physical and sensory qualities of fat in certain food products while providing lower calories [5]. Fat replacers are commonly categorized into three classes depending on their composition: lipid, protein, and carbohydrate-based; each has distinctive functional qualities and could be utilized alone or in combination. For this purpose, gums, pectins, fiber-rich products, carboxymethylcellulose and mal-to-dextrose have been utilized as thickening or stabilizing agents and fat replacers to produce low-fat food [6]. However, the presence of fat replacers may cause unfavorable changes in textural and sensory characteristics. Cold-pressed oil by-products have the potential to lower the amount of fat in food items while maintaining comparable quality and sensory traits in full-fat meals [4,7,8,9]. Therefore, the by-products have come to be used as natural fat replacers and functional ingredients.

Hot peppers (*Capsicum annuum* L.) are a part of Solanaceae family and are widely cultivated around the world, particularly in temperate areas. China is the biggest producer with more than 1,745,000 tons of produce per year, followed by Mexico and Turkey [10]. Hot pepper is widely consumed as a vegetable, a flavoring ingredient, and a natural colorant. Chouaibi and Rezig [11] reported that the hot pepper seeds had 43.6% total carbohydrate, 28.3% protein, 18.4% oil, 6.63% water, and 3.05% ash (on a dry-weight basis). HPSO is rich in bioactive compounds such as phenolics, tocols, phytosterols, and aromatic compounds [12]. Hot pepper is also rich in bioactive carotenoids such as capsaicin and dihydrocapsaicin. After the cold-press process, a by-product rich in polysaccharides, protein, and capsaicin content is revealed. Therefore, HPOB could potentially be used as a fat replacer and an antioxidant agent when producing a low-fat emulsion. In addition, it has been revealed that HPOB has antibacterial, antioxidant [13], and anti-corrosive characteristics [14].

The aim of this study is to make a detailed physicochemical and bioactive characterization of cold-pressed pepper seed oil by-product and to investigate the potential of this by-product as a stabilizing agent in reduced-fat salad dressing. For this purpose, a detailed physicochemical and bioactive characterization of HPOB was performed and rheological and emulsion characteristics such as steady shear, dynamic rheological, 3-ITT rheological properties, emulsion stability, oxidative stability, zeta potential value, and particle size of low-fat salad dressing stabilized by HPOB were studied.

## 2. Materials and Methods

### 2.1. Material

HPOB was purchased from ONEVA Company (Esenyurt, Istanbul). By-products were ground and kept at 10 °C in a closed light-free polypropylene container until analysis. Other ingredients in salad dressing recipes, such as sunflower oil, vinegar, and salt, were purchased from a local market. Sigma-Aldrich provided capsaicin, dihydrocapsaicin, xanthan gum (XG), and egg yolk powder (EYP) (Sigma Chemical Co., St. Louis, MO, USA). The products were kept in a polyethylene container at 25 °C.

### 2.2. Characterization of Hot Pepper Seed Oil By-Products (HPOB)

The oil content of the HPOB sample was analyzed using Soxhlet oil extraction with hexane according to the standard method (AOAC 2003.05) [15]. The moisture, ash, and protein contents of HPOB were measured with standard AOAC method numbers 934.01, 942.05, and 990.03, respectively. The crude fiber determination was carried out using the standard method [15]. 

During the extraction process, HPOB was extracted with a 1:10 (*w:v*) methanol–water mixed solution (8:2, methanol:water) at 25 °C and it was then homogenized using a T-25 Ultra-Turrax (Ika Labortechnik, Stauden, Germany) at 10,000 rpm for three minutes. The mixture was shaken for 2.5 h at 25 °C. After the extraction procedure, the solution was centrifuged at 3311 g for five minutes with a Universal 320 centrifuge (Hettich, Germany), and the supernatant was filtered through a 0.20 µm filter (Sartorius Stedim Biotech, Gottingen, Germany). For further analysis, the extract was kept at −18 °C [16].

According to the modified method proposed by Singleton and Rossi [17], the total phenolic content (TPC) of the methanol extracts was found using Folin–Ciocalteu’s phenol reagent. The TPC of HPOB was measured at 760 nm using a spectrophotometer (Agilent 8453E UV-VIS spectroscopy system). The result was expressed as milligrams of gallic acid equivalent (GAE) per 100 g of dried samples.

The DPPH· free radical scavenging of the methanol extracts was determined according to the method defined by Shimada and Fujikawa [18]. The antioxidant capacity of HPOB was measured at 517 nm with a spectrophotometer (Agilent 8453E UV-vis spectroscopy system) using DPPH·. The results of antioxidant capacity were expressed as a percent of inhibition of the DPPH· radical (%IDPPH).

The antioxidant capacity of the methanol extract was determined according to the CUPRAC (Cupric reducing antioxidant capacity) method [19]. In this assay, 1 mL of CuCl_2_ solution (170.4 mg CuCl_2_.2H_2_O/100 mL water), 1 mL of Neocuproine (Nc) solution (0.156 g Nc/100 mL ethanol), 1 mL of NH_4_Ac (7.708 g NH_4_Ac/100 mL water) solution, and then 1 mL of distilled water was added to 0.1 mL of the extract, respectively. The absorption at 450 nm was determined after 60 min of incubation. The results of antioxidant capacity were reported as mg Trolox per 100 g.

A total carotenoid assay was performed following the method previously proposed by [20] with a slight modification. The extraction of carotenoids was carried out as follows: 1 g of HPOB was dissolved in 50 mL of hexane/acetone/ethanol solution containing 0.1% of BHT and 0.5 g of NaCl. Following 20 min stirring, 15 mL of distilled water was added, and the mixture was stirred for 15 min. The organic phase was separated and filtered. Spectroscopic measurement was performed at 450 nm. The total carotenoid amount was calculated according to Equation (1):

(1) $$Total\;carotenoid\;amount\;(\mu g/g)=\frac{A\times V\times 10^{4}}{E_{1cm}{}^{1\%}\times M}$$

where A is the absorbance at 450 nm, V is the volume of the extract, E_1 cm_^1%^ is the extinction coefficient of β-carotene in hexane, and M is the weight of HPOB. 

GC-MS analysis of capsaicin and dihydrocapsaicin was determined by a method adapted from Peña-Alvarez and Alvarado [21] using a Restec (Bellfonte, PA, USA) Rtx- 5MS fused silica capillary column (30 m × 0.25 mm × 0.25 µm). GC-MS’s operation conditions were as follows: 1 min of heating at 40 °C, and then the temperature raised to 300 °C and kept constant at 300 °C for 10 min. The temperatures of the injection and detection were kept at 260 and 280 °C, respectively. Helium was used as carrier gas at a 1 mL/min flow rate. One microliter of the sample was injected into GC in splitless mode with a 3 min solvent delay. Mass spectra were collected from *m/z* 35 to 650 at an ionization energy of 70 eV. The capsaicin and dihydrocapsaicin were identified and quantified by comparing experimental mass spectra with the mass spectra of these compounds in NIST27 and WILEY27 mass spectra libraries and obtained chromatographic peaks of standard solutions of capsaicin and dihydrocapsaicin.

The specific phenolic compounds were identified using the HPLC technique (HPLC-DAD, Shimadzu Corp., Kyoto, Japan). Before being analyzed in an HPLC system, the TPC extracts were passed through a 0.45 µm membrane filter (Shimad-zu Corp., Kyo-to, Japan). Separations were conducted at 40 °C on a reversed-phase column (Intersil^®^ ODS C-18, GL Sciences, Tokyo, Japan) with a length of 250 mm and a particle size of 5 µm. Solvent A (distilled water with 0.1% (*v/v*) acetic acid) and Solvent B (acetonitrile with 0.1% (*v/v*) acetic acid) were chosen as the mobile phases. Gradient elution was begun with 10% B and held for 2 min. The concentration of solvent B increased from 10% to 30% up to the 27th minutes, from 30% to 90% up to the 50th minute, from 90% to 100% B up to 60 min, and then returned to the starting conditions in 3 min. The discharge rate was one milliliter per minute. Chromatograms were measured between 254 and 356 nm. The phenolic substances were identified and quantified using retention times and standard standards. Individual phenolic content values were reported in mg/L [22].

### 2.3. Salad Dressing Preparation

The salad dressing samples were formulated using the preparation method outlined by Tekin and Karasu [9]. The first step involved dispersing XG (0.35%) in water at 25 °C. The dispersion was then heated to 80 °C, stirred for 20 min, and then an HPOB was added. After adding salt, the mixture was chilled to 25 °C. Following the completion of the material’s dissolution, the XG was completely hydrated by being stirred for 6 h at 1000 rpm in a magnetic mixer. Using Ultra Turrax (Daihan, HG15D) at 10,000 rpm for 3 min, homogenization was performed after adding sunflower oil and EYP (3%). Salad sauce was eventually acquired and pasteurized for 10 min at 65 °C. After homogenization and cooling to 25 °C, salad dressing samples were put into brown flasks [23]. The same procedures also prepared the control salad dressing sample. Control samples (C1 and C2) were formulated with 10% and 30% sunflower oil, respectively. All control samples contained 0.35% of XG, 3% of EYP, 0.35% of XG, and 3% of EYP (Table 1).

### 2.4. Analysis of Salad Dressing

#### 2.4.1. Rheological Analysis

The rheological analyses of salad dressings were evaluated at 25 °C using a temperature-controlled rheometer (MCR 302; Anton Paar, Sydney, NSW, Austria); specifically, the flow behavior properties, dynamic rheology, 3ITT rheological properties, and emulsion stability (thermal loop test) were evaluated. 

Utilizing a parallel plate configuration and a spacing of 0.5 mm between the rheometer probe and the sample plate in the range of 0–100 shear rate (1/s), the flow behavior and rheological characteristics of the salad dressings were evaluated. To support the rheometer measurement plate while the temperature was being reached and analysis was being conducted, 2 g of the sample was inserted. The values of shear stress that corresponded to the shear rate were first determined. The parameters of flow behavior and rheological properties were then assessed using the Power Law model and nonlinear regression.

(2) $$\tau ={K\gamma }^{n}$$

In Equation (2), τ shows shear stress (Pa), K—consistency index (Pa·s^n^), γ—shear rate (1/s), and *n*—the flow behavior index.

The samples were subjected to a dynamic rheological study on parallel plates. The amplitude sweep test was first carried out with a strain value of 0.1% in order to assess the linear viscoelastic region (LVR). The frequency sweep measurement was conducted in LVR between 0.1 and 64 (ω) angular speeds and 0.1 to 10 Hz. The storage modulus (G′) and loss modulus (G″), in addition to rotational velocity and frequency, were determined. Parameters unique to complicated rheological properties were evaluated using the Power Law model and nonlinear regression [24].

(3) $$G\prime =K\prime {\left(\omega \right)}^{n\prime }$$

(4) $$G\prime \prime =K\prime \prime {\left(\omega \right)}^{n\prime \prime }$$

In Equations (3) and (4), G′ (Pa) is the storage modulus, G″ (Pa) is the loss modulus, ω is the angular velocity value (1/s), and K′, K″, and consistency index values *n*′ represent the flow behavior index values.

The 3-ITT rheological characteristics for the salad dressing samples were calculated to be a steady shear rate of 0.5 s^−1^ and a varying shear rate of 150 s^−1^, respectively. As the values were selected, the LVR of the samples was taken into consideration, and the LVR of the samples stops at 50 s^−1^. Salad dressing samples were subjected during the first time period for 100 s at a very low shear rate (0.5 s^−1^). For 40 s during the second time period, 150/s was exposed to the prescribed shear force. By subjecting the samples to a modest shear rate in the first time interval during the third time period, the dynamic rheological behavior in the second time interval was evaluated.

(5) $${\left[\frac{G\prime -G_{e}}{G_{0}-G_{e}}\right]}^{1-n}=(n-1)kt+1$$

In Equation (5), G′ represents the storage module in (Pa), k is the thixotropic rate constant, G_0_ is an initial storage modal value (Pa) in the third time interval, and G_e_ is the equilibrium storage modulus (the product completely recovers itself) [25].

Tekin and Avci [26] first reported that the thermal loop test was used to evaluate the emulsion stability of oil in water emulsions exposed to eleven thermal cycles at high (from 23 to 45 °C) and low (from 5 to 23 °C) temperatures. A thermal loop test is a suitable method to detect structural changes and replicate temperature differences in production, distribution, storage, and transport in a short time. Different amounts of heat were applied to the emulsions in cycles. The strain value and angular frequency (ω) were set to 0.5% and 10 Hz, respectively. The cooling and heating rates were arranged as 11 K/min. The maximum values of all cycles were found using the internal loop and the rheometer software. The change of modules from cycle to cycle reflects the structural or morphological changes induced by temperature stress. As a result, the temperature loop test can be used to estimate oil stability in water emulsions such as salad dressing and mayonnaise.

#### 2.4.2. Zeta Potential (ζ) and Particle Size Measurement

The oil particle size distributed in the continuous phase of the salad dressing samples was calculated using a Zeta (ζ-) potential and particle size meter (Nanosizer, Malvern Instruments, Worcestershire, UK) with electrophoresis and a dynamic light scattering device. The samples were first diluted 500 times with ultrapure water until the measurement was reached, and then homogenized in an ultrasonic water bath in a few seconds. The procedure was then repeated in duplicate for each sample using the Zeta potential measurement, and the averages and standard deviations were calculated.

#### 2.4.3. Optical Microscope Observation

The morphology of the emulsions before and after the thermal loop test was examined using a light microscope (Olympus BX41, Tokyo, Japan) at 100× magnification. First, a single droplet of sauce sample was put on a microscopy glass and covered with a coverslip. The emulsion stability was then fully evaluated by observing various slide regions.

#### 2.4.4. Oxidative Stability

The oxidative stability of the salad dressing samples was determined using the OXITEST Device (Velp Scientifica, Usmate, MB, Italy) as described by Aksoy and Tekin-Cakmak [8]. The samples were subjected to an accelerated oxidation test using the OXITEST device. A sample (20 g) was put in the device’s receptacle. At 6 pressure and 90 °C, the rapid oxidation test was performed. The induction period number (IP) was considered when assessing redox stability.

The detection of volatile lipid peroxidation compounds was performed by solid-phase microextraction-gas chromatography-mass spectrometry (SPME-GC-MS) according to Shi and Bucknall’s [27] method with a slight modification. In brief, hot dressing samples were transferred to 20 mL headspace vials. The vials were heated to 60 °C and equilibrated for 15 min. Next, SPME fiber (30/50 μm DVB/ CAR/PDMS SPME fiber assembly, Supelco, Bellefonte, PA, USA) was exposed to vial headspace for 30 min at 60 °C. Then, the SPME fiber was inserted into the GC injection port at 250 °C for 5 min. The operation conditions in GC-MS were as follows: 3 min of heating at 40 °C, and then the temperature raised to 90 °C at 5 °C/min, and to 230 °C at 10 °C/min. Helium was used as carrier gas at a 1 mL/min flow rate. The ion source was EI, and the temperature was set at 200 °C. Mass spectra were collected from *m/z* 35 to 650 at an ionization energy of 70 eV. By matching experimental mass spectra to the mass spectra of these compounds in the NIST27 and WILEY27 mass spectra libraries, the lipid peroxidation products were found.

#### 2.4.5. Statistical Analysis

All samples were prepared in duplicate, and each replication received three parallel measurements. The data’s standard deviation and mean value were presented. For statistical analysis, the STATISTICA software application (Stat Soft Inc., Tulsa, UK) was used. Duncan’s analyses compared the factors’ mean values at a confidence interval of 0.05. Because of the applied rheological research, power-law and second-order structural kinetic model parameters were produced using nonlinear regression analysis. STATISTICA software was used to perform the nonlinear regression analysis (Stat Soft Inc., Tulsa, UK).

## 3. Results and Discussions

### 3.1. Characterization of HPOB

The moisture, protein, oil, crude fiber, ash, and carbohydrate amounts of HPOB were 6.69%, 20.2%, 11.2%, 31.9%, 3.83%, and 57.7%, respectively (Table 2), indicating that HPOB was rich in protein and fiber content. Unfortunately, the number of studies offering the composition of HPOB is scarce. In a study conducted by Yılmaz and Hüriyet [28], defatted pepper cold-pressed cake’s composition was 8.89% moisture, 19.3% protein, 2.86% oil, and 6.28% ash. The lower oil amount in this study may be associated with solvent extraction following cold-pressing. The moisture, protein, ash, and carbohydrate content of defatted red pepper seed flour were 10.7%, 26.0%, 4.10%, and 55.1%, respectively [10]. The amount of crude fiber was 34.9% in paprika seed flour [29]. Since the variety, maturation stage, and growing conditions of hot pepper affect the final quality and composition, small differences may be present in the physicochemical properties of the hot pepper studied in the literature. 

The TPC, IDPPH (%), and CUPRAC values of HPOB were determined as 317.4 mg GAE/100 g, 81.8%, and 6952.8 mg Trolox/100 g, respectively. The capsaicin (CAP), dihydrocapsaicin (DHC), and total carotenoid content were 175.8 mg/100 g, 71.0 mg/100 g, and 106.3 µg/g, respectively. To the best of our knowledge, it is hard to find studies demonstrating the quantity of CAP and DHC in HPOB. In a study conducted by Yang and Mandal [30], CAP and DHC amounts of hot pepper seed oil were 0.16 mg/mL and 0.07 mg/mL, respectively. Moreover, Chouaibi and Rezig [11] determined that TPC, total carotenoids, CAP, and the DHC contents of red pepper seed oils extracted via cold-pressing were 8.27 mg/100 g, 4.39 mg/100 g, 3.45 mg/kg, and 2.81 mg/kg, respectively. 

The antioxidant potential of hot pepper powder obtained from the CUPRAC method was 3626 mg Trolox/100 g, and the TPC of the hot pepper powder was 1682 mg GAE/g [31]. The presence of a wide range of hot pepper varieties leads to compositional differences in the quantities of bioactive compounds. HPOB contained all standard phenolic compounds except for 4-hydroxybenzoic acid and m-coumaric acid. In this study, myricetin (87.61 μg/g dry weight) and gallic acid (66.02 μg/g dry weight) were identified as abundant individual phenolic components in HPOB.

### 3.2. Rheological Analyzes

#### 3.2.1. Flow Behavior Rheological Properties

**Figure 1:**
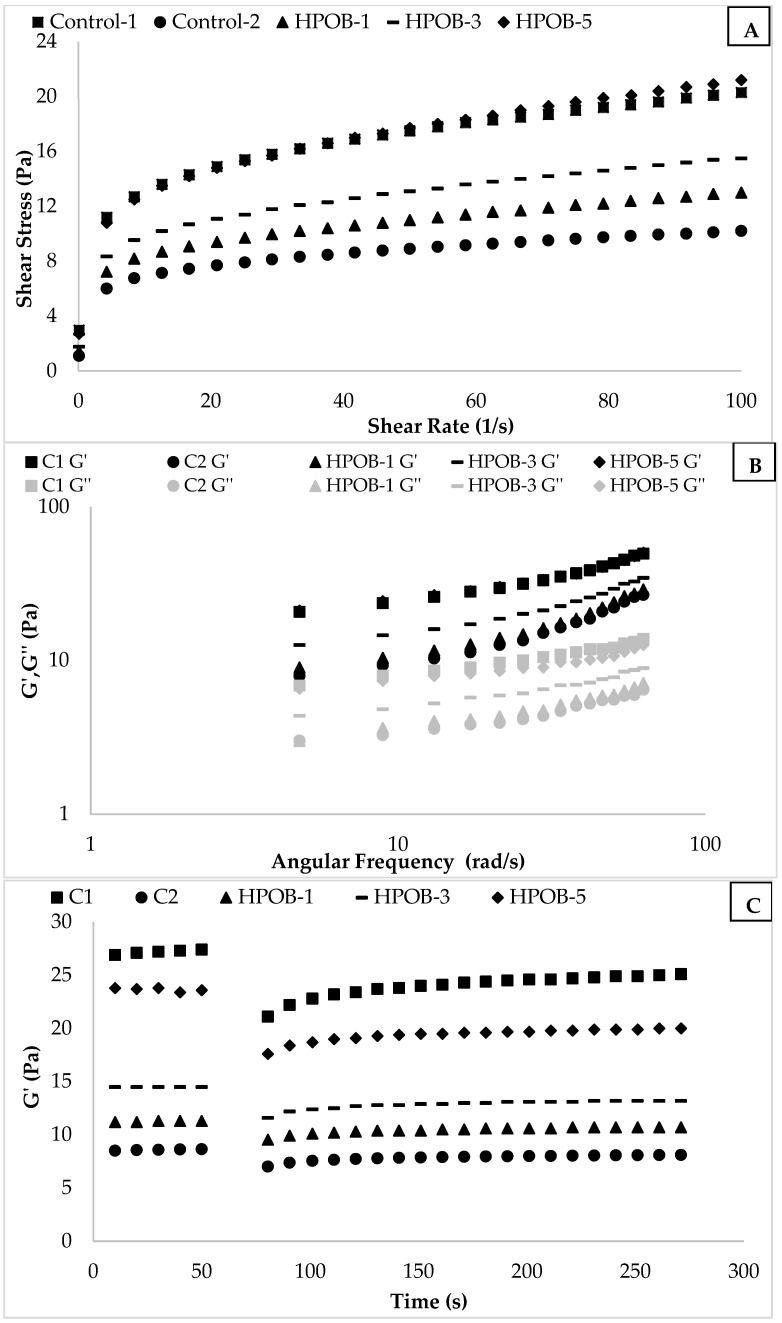
The rheological properties of salad dressing samples ((**A**): Steady shear rheological properties, (**B**): dynamic rheological properties, (**C**): 3-ITT rheological properties).

Figure 1A shows the steady shear rheological properties of the samples. The viscosity values of the salad dressing samples decreased with increasing shear rate, as explained by the shear rate versus the shear stress curve’s decreasing slope with increasing shear rate. This flow pattern is a common rheological characteristic of emulsions that resemble salad dressings. The weak bonds between molecules and the weakened contact between the components caused by the applied shear force may be the cause of the viscosity decrease with the increasing shear rate [32]. In addition, all samples exhibited non-Newtonian flow behavior, a specific rheological property of salad-dressing-like emulsions [33,34]. 

The steady shear rheological analysis data were applied to the Power Law model to assess a numerical comparison of a sample’s flow behavior characteristics. The Power Law model characteristics of salad dressing samples are indicated in Table 3. The R^2^ values in the table are greater than 0.97, showing that the Power Law model effectively modeled the steady shear of the rheological properties of the samples. The K values of the samples varied according to the product compositions and were found to be 4.10–7.79 Pa.sn. While C1 (the low-fat control sample) and C2 (the high-fat control sample) had K values of 4.10 and 7.79 Pa.s^n^, respectively, the K values of low-fat samples enriched with HPOB (from 1% to 5%) rose from 4.82 Pa.s^n^ to 7.25 Pa.s^n^. With the addition of HPOB (5%), the K value of the low-fat salad dressing sample was similar to the K value of the high-fat salad dressing sample. One of the most significant variables influencing the rheological characteristics of emulsions such as salad dressing is the oil ratio.

The addition of HPOB greatly raised the K value of the low-fat control sample. This finding showed that the weakened structure caused by the decrease in oil content could be compensated for by the inclusion of HPOB. The water-holding property of the protein and fibers in the HPOB structure and the various interactions between protein and polysaccharide may have reduced the mobility of the continuous phase and caused the samples to reach the desired consistency level.

The n values of the samples were between 0.200 and 0.231. The n value of all the samples was less than 1, indicating that the samples exhibited non-Newtonian flow behavior. For salad dressing samples, the n value is expected to be close to 0, that is, to have a strong pseudoplastic flow property. Therefore, it can be said that the n value of most of the salad dressing samples is lower than n: 0.25; that is, it is close to the desired values [35,36]. As predicted, a negative association was found between k and n values, which can be explained by the previously mentioned drop in the n value, rise in pseudoplastic flow behavior, and creation of a tight structure. The addition of HPOB greatly raised the K values of the samples with a reduced oil content, according to this study. Oil plays an essential role in maintaining the consistency of O/W emulsions. Although calorie reduction was achieved by reducing the amount of fat, consistency and consumer preference decreased. However, the mobility of the continuous phase was controlled, and the consistency was regained thanks to the water holding of fiber and protein of HPOB. 

#### 3.2.2. Dynamical Rheological Properties

Figure 1B represents the dynamic rheological behavior of salad dressing samples. The G′ values of all samples were higher than the G′ values in all frequency ranges, as indicated in Figure 1B. Furthermore, the G′ and G″ values of the samples increased at a diminishing rate as the angular velocity increased; in other words, the slope of the angular frequency versus G′ and G″ graph dropped. This is typical of the movement behavior of viscous solids. Since salad dressings are products with a viscoelastic solid character, each salad dressing sample produced has the expected properties [9].

Table 3 shows the K′, K″, n′, and n″ values found using nonlinear regression with the Power Law model. As shown in Table 3, R^2^ values were relatively high (R^2^ > 0.97), suggesting that the model can effectively describe the samples’ dynamic rheological behavior. The salad dressing samples’ K′ and K″ values were estimated to be 2.21–10.47 Pa.s^n^ and 1.71–4.76 Pa.s^n^, respectively, and these values differed depending on the composition. In all examples, the K′ value is greater than the K″ value, indicating that viscous solid behavior was observed. In addition, the sample of C-1 (30% oil) and the sample of HPOB-5 (10% oil and 5% HPOB) have the same K′ (10.47 Pa.s^n^). In this case, C-1 and HPOB-5 samples have the same viscoelastic solid behavior, and it is seen that 20% oil content is provided with 5% HPOB. The n′ and n″ values changed between the values of 0.359–0.589 and 0.209–0.311, respectively. The n′ and n″ values are another indication of the viscoelastic solid character of the samples; these values approach 0, indicating that the solid character of the samples is more dominant. The enrichment with HPOB significantly increased K′ and decreased n′. Therefore, a viscoelastic solid character, which is the desired structure for salad-dressing-like emulsions, could be increased by the enrichment of HPOB at a lower oil content. 

#### 3.2.3. The 3-ITT Properties

The 3-ITT, a crucial test that demonstrates the impact of an abrupt force or deformation on food’s rheological characteristics, can mimic the impact of motions such as shaking and pressing on food in everyday life [25]. The evolution of the G value for the salad vinaigrette samples is depicted in Figure 1C. The degree of sample recovery following an abrupt displacement is depicted in Figure 1C and is shown to vary depending on the applied shear rate. The capacity of each sample to restore itself diminishes as the deformation value rises. The G′ values of the salad dressing samples changed over time, as shown in Figure 1C. As seen in Figure 1C, the degree of recovery of the sample due to a sudden deformation varies depending on the applied shear rate. The ability of each sample to restore itself diminishes as the deformation value increases. In other words, as the samples exposed to a higher shear rate underwent more structural deformation, a significant decrease was observed in the G′ value of the product, and it became impossible to reach the initial G′ value again.

The parameters relating to structural recovery need to be obtained more accurately compared to the samples’ recovery pattern. Table 3 indicates that the data obtained from the 3-ITT test were modeled with the second-order structural kinetic model and the obtained G_0_′, G_e_′, and k values. Since each G_0_′ and G_e_′ value is different in Table 3, the comparison of the recovery degree of the products is based on the ratio of these two values (G_e_′/G_0_′) [37]. In Table 3, G_e_′/G_0_′ values vary between 1.213 and 1.303. It was seen that the effect of both the oil content and the by-product amount on G_e_′/G_0_′ values was significant (*p* < 0.05). Considering that the G_e_′/G_0_′ values give an idea about the degree of recovery of the product, it is an expected result to increase with the increase in the amount of oil, but it is also a positive result that it increases with the addition of HPOB.

Another parameter obtained in the 3-ITT test data is the k value. The k value indicates the recovery rate of the product after the sudden deformation. This situation is related to the structure of the product. If no permanent deformation is observed in the product, the recovery speed of the product will be higher. In such a rapid way, the value of G′ will reach its desired level, that is, its initial value. The k values obtained for salad dressings were significantly affected by the product type and the applied shear rate value (*p* < 0.05). The value of k × 1000 varied between 43.02 and 60.61 (Table 3). As shown in Table 3, the value of k increased as the amount of by-product (HPOB) increased in low-fat salad dressings. The 3-ITT test concluded that low-fat salad dressing, whose recovery level is similar to full-fat salad dressing, could be produced with HPOB enrichment. 

#### 3.2.4. Emulsion Stability

**Figure 2:**
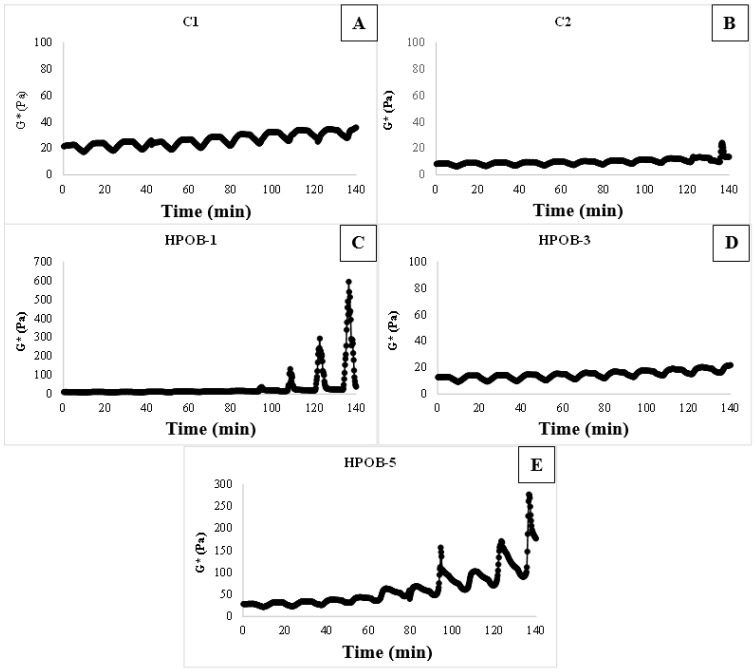
Emulsion stability test via thermal loop test (The change in G* values for samples: (**A**): C1: samples contained 30% oil. (**B**): C2: samples contained 10% oil (low-fat salad dressing sample), (**C**): samples contained 1% HPOB and 10% oil, (**D**): samples contained 3% HPOB and 10% oil, (**E**): samples contained 5% HPOB and 10% oil).

One of the most crucial quality criteria of O/W emulsion is emulsion stability because salad dressings with poor emulsion stability may experience phase separation on the top during storage. According to [26], the thermal loop test used differences in G* to determine the stability of an emulsion; larger changes in G* indicated emulsion instability in thermally generated cycles. By using 10 separate loops, the high temperature (25–45 °C) loop experiments for salad dressing samples revealed changes in G* values, as shown in Figure 2A,B. In the high-temperature thermal loop experiments, G* dropped as the temperature rose, as shown in Figure 2.

After the third cycle, a significant change in G* was seen in the low-fat control sample 1 (Figure 2A), suggesting that the low-fat control emulsion had reduced emulsion stability. Because oil droplet packing percentage raises and lowers creaming/sedimentation rates, raising the oil phase volume fraction usually improves emulsion stability [38]. 

Sample HPOB-3, as seen in Figure 2D, demonstrated little change in the G* number, demonstrating excellent emulsion stability. But between 25 and 45 °C, a significant rise in G* was seen in the HPOB-5 sample. This was accounted for by a number of factors, including a change in the solubility and viscosity of XG in the aqueous phase and a change in viscosity in the disseminated phase (Figure 2E). Some instability processes occurred as a consequence. Each sample was examined to look for phase separation after 10 heat cycles. Only the low-fat control and HPOB-1 exhibited phase separation, demonstrating a correlation between the emulsion stability test findings from the thermal loop test and the ocular analysis.

### 3.3. Zeta (ζ) Potential, Particle Size, and Light Microscope Images

The Zeta (ζ) potential of salad dressings is a significant indicator of whether they can be steady for a long period. In other words, as the ζ potential number moves away from zero, the system acquires a negative or positive charge, which indicates the sample’s extended stability [39]. Table 3 indicated that ζ potential values of C1, C2, HPOB-1, HPOB-3, and HPOB-5 samples were determined as −40.17 ± 1.50 mV to −30.33 ± 2.19 mV. Indicating that all samples showed high ζ potential values. The formation of a negative charge is due to the bonding of the negative charges around the oil droplets with polysaccharides through hydrophobic bonds. The fact that the ζ potential values of the salad dressing samples containing cold-pressed hot pepper seed oil and their by-products were found in the range of −29.33 to −35.67 mV might have indicated that these formulations can remain stable for a long time.

Particle size (d_32_) is a critical factor in determining the emulsion stability of salad dressing samples. The particle size (d_32_) of C1, C2, HPOB-1, HPOB-3, and HPOB-5 samples were found to be 3.44 ± 0.16 µm, 3.94 ± 0.24 µm, 2.04 ± 0.48 µm, 2.82 ± 0.13 µm, 2.63 ± 0.35 µm, and 1.72 ± 0.05 µm, respectively. As seen in Table 3, the particle sizes of the samples with the high absolute value of ζ potential were also lower. The reduction in ζ potential was linked to a decrease in mobile phase activity and easier emulsifier and protein localization around oil molecules. Researchers emphasized that another important effect affecting particle diameter is negatively charged polysaccharides. The cold-pressed oil by-products could be used as a stabilizing agent to improve the rheological properties of low-fat products.

**Figure 3:**
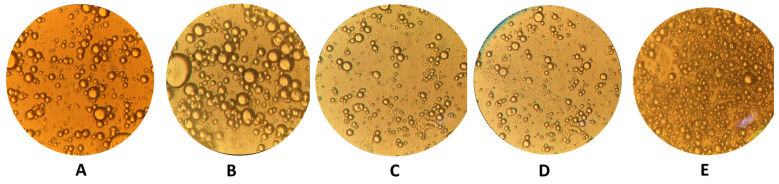
The light microscopic images of salad dressing samples ((**A**): C1. (**B**): C2. (**C**): HPOB 1. (**D**): HPOB 3. (**E**): HPOB 5).

Figure 3 indicates the light microscope images of the salad dressing samples. Samples containing HPOB have lower droplet sizes, although they contain less oil. These results were in agreement with the emulsion stability analysis and zeta sizer measurements. HPOB may have prevented the oil droplets from coming together by reducing the mobility in the aqueous phase. Emulsion stability, zeta sizer measurements, and light microscopy data showed that HPOB could improve emulsion stability in fat-reduced salad dressing.

### 3.4. IP Value and Oxidative Volatile Formation

The oxidation stability of low-fat salad dressing samples containing HPOB (1–3 and 5%), as well as full-fat and low-fat control samples (C1 and C2), was measured using the OXITEST Device. IP (induction period) values were used to assess the oxidative stability of the materials. Table 4 shows that the IP values of hot pepper seed oil were 7.03 h. In addition, the IP values of salad vinaigrette samples at 90 °C varied between 2.58 and 8.33 h. (Table 4). The IP values of the salad dressing samples containing HPOB (between 6.33 h and 8.33 h) were higher than the IP values of the control samples (3.20 h and 2.58 h), indicating that HPOB improved the oxidative stability of the salad dressing samples significantly.

The volatile components formed due to oxidation were the highest in the reduced-fat control sample (C2). The oil fraction ratio in oil/water emulsions is a critical quantity influencing oxidation stability. Oxidation stability reduces as the oil fraction percentage drops [40]. In the samples containing HPOB, fewer oxidative volatile components were formed than in the oily control sample and reduced-fat control sample (C1 and C2, respectively). In particular, the formation of medium- and short-chain aldehydes used as oxidation markers was significantly reduced in samples containing HPOB. There is an agreement between the results obtained with the formation of oxidative volatile components of hexanal, nonanal, and 1-octene-3-ol and the IP values. Both results showed that HPOB-containing salad dressing samples had higher oxidative stability than the control, although the oil was reduced. This can be explained by the capsaicin, phenolic, and protein content of HPOB. The localization of HPOB derivative phenolics could explain the increase in oxidative stability of salad dressings at the oil–water interface. Lee and Han [41] studied the thermal-oxidative stability of lard, soybean oil, and hot pepper seed oil for 0–3 days at 100 °C. The capsaicin, dihidrocapsaicin, and total capsaicin content of HPOB were determined as 17.58 mg/100 g, 7.10 mg/100 g, and 24.67 mg/100 g. According to Yang and Mandal [30], red pepper seed oil can be used instead of soybean oil to avoid thermal oxidation during deep-frying, and thermal acidification can be further stopped by adding capsaicin or tocopherol as antioxidants.

Lee and Han [41] reported that hot pepper seed oil had an antioxidative effect due to its antioxidants, such as capsaicin and α-tocopherol, so capsaicin inhibited lipid oxidation in lard but not in soybean oil. Besides capsaicin and phenolic compounds, proteins have oxidation-retarding properties in oil/water emulsions. Proteins have the potential to delay oxidation because of their ability to scavenge free radicals and volatile oxidative compounds containing carbonyl groups such as aldehydes and ketones [40]. The high protein content in HPOB may have effectively reduced oxidation by both mechanisms. In conclusion, the results of this study suggested that HPOB could improve the thermal-oxidative stability of reduced-fat salad dressing samples in addition to improving rheological properties.

## 4. Conclusions

Bioactive compounds, fiber, and protein-rich by-products are produced during cold-pressed oil manufacturing. Converting this by-product into a high-value supply is a critical problem for cold-pressed oil producers. The rheological properties, emulsion, and oxidative stability enhancement potential of HPOB in reduced-fat salad dressing were examined in this study. HPOB contained high phenolic compounds, capsaicin, dihydrocapsaicin, fiber, and protein content. The pseudo-plastic, viscoelastic solid, and recoverable character of low-fat salad dressings was improved by the enrichment of HPOB. The results of the thermal loop test, zeta potential, and particle size distribution showed that enrichment with HPOB could increase the samples’ emulsion stability and microstructural properties. In addition, the IP values of the samples enriched with HPOB increased, and the amount of volatile oxidative components decreased. According to the findings of this research, HPOB could be used to enhance the rheological characteristics and oxidative stability of low-fat salad dressing, and a low fat salad dressing with excellent emulsion stability could be produced with the addition of 3 % HPOB.

## Figures and Tables

**Table 1:** Ingredients of salad dressing samples.

	Water (%)	Oil (%)	By-Product (%)	Vinegar (%)	EYP (%)	Salt (%)	XG (%)
C1	58.65	30	-	7	3	1	0.35
C2	78.65	10	-	7	3	1	0.35
HPOB-1	77.86	10	1	7	3	1	0.35
HPOB-3	75.65	10	3	7	3	1	0.35
HPOB-5	73.65	10	5	7	3	1	0.35

HPOB: Hot pepper seed oil by-products. C1: samples contained 30% oil. C2: samples contained 10% oil (low-fat salad dressing sample). The different lowercase letter in the same line indicates significance between samples (*p* < 0.05). All ingredients are expressed as a percentage (*w/w*).

**Table 2:** Physicochemical properties of HPOB.

HPOB	
Moisture (%)	6.69 ± 0.03
Protein (%)	20.25 ± 0.18
Oil (%)	11.24 ± 0.13
Carbonhydrate (%)	57.72 ± 0.04
Crude fiber (%)	31.91 ± 0.28
Ash (%)	3.83 ± 0.07
TPC (mg GA/100 g)	317.5 ± 19.17
I_DPPH_ (%)	81.87 ± 0.01
CUPRAC (mg Trolox/100 g)	6952.8 ± 170.01
Capsaicin (µg/g)	175.8 ± 1.65
Dihidrocapsaisin (µg /g)	71.00 ± 1.46
Total carotenoids (µg/g)	106.3 ± 2.31
Fatty acid composition	
C12:0	0.006 ± 0.00
C16:0	13.51 ± 0.36
C18:0	2.542 ± 0.03
C18:1	6.87 ± 0.06
C18:2	73.29 ± 0.41
C18:3	2.75 ± 0.03
C20:0	0.045 ± 0.003
Phenolic Compounds	μg/g of Dry Weight
Gallic acid	66.02 ± 0.72
Protocatechuic acid	15.05 ± 0.35
Catechin	16.73 ± 0.51
4-Hydroxy-benzoic acid	nd
Syringic acid	3.85 ± 0.50
Ellagic acid	41.85 ± 0.61
m-Coumaric acid	nd
o-Coumaric acid	8.03 ± 0.37
Chrysin	1.60 ± 0.03
Caffeic acid	17.19 ± 0.32
p-Coumaric acid	5.88 ± 0.02
Ferulic acid	6.56 ± 0.04
Myricetin	87.61 ± 0.43
Quercetin	48.09 ± 0.52
Kaempferol	30.57 ± 0.15
Chlorogenic acid	36.87 ± 0.62
Rutin	29.52 ± 0.18
Sinapic acid	3.30 ± 0.02

HPOB: hot pepper seed oil by-products, IDPPH: inhibition percentage of the DPPH· radicals. TPC: total phenolic compounds. nd: not detected.

**Table 3:** Rheological parameters, ζ potential and particle size distribution of salad dressing samples.

	C1	C2	HPOB-1	HPOB-3	HPOB-5
Steady Shear Rheological Parameters					
K (Pas^n^)	7.79 ± 0.21 ^a^	4.10 ± 0.07 ^e^	4.82 ± 0.15 ^d^	5.79 ± 0.06 ^b^	7.45 ± 0.17 ^a^
n	0.208	0.200	0.214	0.221	0.231
R^2^	0.993	0.982	0.987	0.991	0.995
Dynamic Rheological Parameters					
K′ (Pas^n^)	10.47 ± 0.06 ^a^	2.21 ± 0.08 ^e^	2.72 ± 0.15 ^d^	5.18 ± 0.12 ^b^	10.47 ± 0.02 ^a^
n′	0.359	0.589	0.553	0.440	0.359
R^2^	0.978	0.978	0.972	0.974	0.978
K″ (Pas^n^)	4.40 ± 0.03 ^b^	1.71 ± 0.04 ^f^	1.81 ± 0.02 ^e^	2.74 ± 0.03 ^c^	4.76 ± 0.05 ^a^
n″	0.263	0.300	0.311	0.269	0.209
R^2^	0.991	0.967	0.972	0.972	0.946
3-ITT Rheological Parameters					
G_0_′	19.80	6.39	8.99	10.69	15.73
G_e_′	25.81	8.24	10.91	13.44	20.31
G_e_′/G_0_′	1.303	1.290	1.213	1.257	1.291
K × 1000	60.61	53.52	43.02	52.73	57.39
R^2^	0.997	0.998	0.994	0.995	0.997
ζ Potential and Particle Size Distribution					
ζ-potential (mV)	−40.17 ± 1.50 ^c^	−28.97 ± 2.17 ^bc^	−29.33 ± 0.68 ^a^	−32.20 ± 0.59 ^a^	−35.67 ± 0.55 ^b^
d_32_ (µm)	3.44 ± 0.16 ^a^	4.94 ± 0.24 ^a^	4.83 ± 0.15 ^bc^	4.44 ± 0.08 ^cd^	4.22 ± 0.13 ^b^
PdI	0.95 ± 0.04 ^a^	0.85 ± 0.22 ^a^	0.87 ± 0.17 ^bc^	0.92 ± 0.15 ^ab^	0.93 ± 0.26 ^c^

HPOB: Hot pepper seed oil by-products. C1: samples contained 30% oil. C2: samples contained 10% oil (low-fat salad dressing sample). The different lowercase letter in the same line indicates significance between samples (*p* < 0.05).

**Table 4:** Oxidative volatile compounds and IP value.

OVC and IP Value	Sample									
	C1		C2		HPOB1		HPOB3		HPOB5	
IP value (h)	3.20 ± 0.04 ^d^		2.58 ± 0.05 ^e^		6.33 ± 0.03 ^c^		7.18 ± 0.12 ^b^		8:33 ± 0.06 ^a^	
	Storage time (days) Peak area (TIC units·10^−6^)									
	0.	7.	0.	7.	0.	7.	0.	7.	0.	7.
Aldehydes										
Pentanal	nd	0.33		0.34		nd		nd	0.21	nd
Hexanal		2.77		6.42		1.52		0.85		0.75
2-Heptenal		0.45		0.43		nd		nd		nd
Octanal		0.60		0.82		nd		0.28		1.57
2-Octenal	0.61	0.26		nd				0.38		nd
Nonanal		0.78		1.17	0.29	0.86		0.74	0.27	0.69
2-Nonenal		nd		nd		0.72		nd		nd
2-Decenal		nd		nd		0.81		nd		nd
Decanal		0.17		nd		nd		nd		nd
2,4-Decadienal		nd		nd		nd		0.47		nd
Alcohol		nd								
1-Pentanol		nd		0.31		nd		nd		nd
1-Heptanol		nd		0.24		0.32		0.55		0.65
1-Octene-3-ol		0.99		1.52	0.26	0.88	0.31	0.71		0.64
1-Penten-3-ol		nd		nd		nd		nd		nd
Ketone										
2-Heptanone		nd		0.27		0.35		nd		nd
3-Heptanone-5-methyle		nd		0.23		nd		nd		0.99
Hydrocarbons										
Pentane		nd		nd		nd		nd		0.38
Heptane		nd		nd		nd		nd		nd
Octane		nd		nd		1.98		nd		nd
1-3-Hexadiene		nd		0.48		nd		nd		nd
Furan										
2-Pentylfuran		1.41		1.51		0.91		0.83		0.89
2-Ethylfuran		nd		0.13		nd		nd		nd
Acids										
Hexanoic acid		nd		nd		nd		nd		nd
Propanoic acid		nd		nd		0.38		nd		nd

HPOB: Hot pepper seed oil by-products. C1: samples contained 30% oil. C2: samples contained 10% oil (low-fat salad dressing sample). The different lowercase letter in the same line indicates significance between samples (*p* < 0.05).

## Data Availability

The data presented in this study are available on request from the corresponding author.
